# Editorial: Deep learning with limited labeled data for vision, audio, and text

**DOI:** 10.3389/frai.2023.1213419

**Published:** 2023-06-13

**Authors:** Marko Orescanin, Leslie N. Smith, Saurabh Sahu, Palash Goyal, Sujit Rokka Chhetri

**Affiliations:** ^1^Computer Science Department, Naval Postgraduate School, Monterey, CA, United States; ^2^Naval Center for Applied Research in Artificial Intelligence (NCARAI), U.S. Naval Research Laboratory, Washington, DC, United States; ^3^Amazon, Cambridge, MA, United States; ^4^Amazon.com LLC, Sunnyvale, CA, United States; ^5^Palo Alto Networks, Santa Clara, CA, United States

**Keywords:** deep learning, few shot learning, semi-supervised, neural network, active learning, semantic segmentation

Deep learning has made significant strides in computer vision, audio, and natural language processing over the past decade. However, the success of deep learning models is often dependent on the availability of labeled data. Labeled data, where each data point is accompanied by a corresponding label, is crucial for training supervised deep learning models. Unfortunately, in many real-world applications, acquiring labeled data can be expensive, time-consuming, and sometimes even impossible. Hence, it is imperative that methods be developed to enable deep learning work effectively where limited labeled data is available. In this issue, we present works that tackle the problem of learning with limited data having real-world impact in various fields such as sports and medicine. The articles talk about methods that approach the problem from different perspectives and offer innovative solutions to develop state-of-the-art models.

In Jersey number detection using synthetic data in a low-data regime (Bhargavi et al.) generate synthetic datasets to address the problem of data scarcity when training models to identify players in American football. They used pre-trained models to identify potential image areas with jersey numbers and ended up with a limited size dataset with high class-imbalance after human annotations. To counter these issues, the authors created a synthetic dataset leveraging various image augmentation techniques and pre-existing image datasets. Using these methods, the authors showed a significant improvement in model performance when applied in the wild.

In “*Active learning for data efficient semantic segmentation of canine bones in radiographs*”, Moreira da Silva et al. address the challenge of efficiently selecting datapoints for annotation that would maximize the model's performance and reduce the redundancy in human annotations. Leveraging a model trained with a small, annotated training set, the authors selected datapoints from a larger unlabeled set to be given for human annotation in a step-wise approach where the selection criteria aimed to favor samples that are either very diverse from the existing training set or for which the trained model is not very confident on its predictions. The authors showed that smartly selecting samples for annotation can lead to high performing models trained using only a fraction of the data, with reduced time complexity.

Finally, Smith and Conovaloff explore the paradigm of one-shot learning where the assumption is that there is only labeled sample per class available for training. The authors rely on semi-supervised learning techniques to learn from unlabeled data-points. Specifically, they employ the techniques of pseudo-labeling and self-training and pick confidently labeled samples from unlabeled dataset to be added to their labeled training set. Unlike active learning there was no human effort involved at all. Finally, the authors show that selecting an iconic datapoint as the prototype training example for one shot training can allay the need of labeling a large number of samples for training deep neural networks.

We thank the authors, reviewers, and editors for all their efforts in making this issue a reality. We hope our readers enjoy the articles and get inspired to explore this field of deep learning.

## Author contributions

All authors listed have made a substantial, direct, and intellectual contribution to the work and approved it for publication.

